# Repaying Kindness and Resilience: Cultural Insights for Nursing Care in Chinese Breast Cancer Recovery

**DOI:** 10.1002/nop2.70319

**Published:** 2025-10-03

**Authors:** Weiwei Liu, Meng Liu, Xiaofeng Chen

**Affiliations:** ^1^ School of Sociology Beijing University of Technology Beijing China; ^2^ College of International Education and Social Development Zhejiang Normal University Jinhua Zhejiang China; ^3^ College of Economics and Management Zhejiang Agriculture and Forestry University Hangzhou Zhejiang China

## Abstract

**Aim:**

To explore the role of **repaying kindness** (*bào'ēn*), a culturally embedded coping mechanism, in the recovery of breast cancer survivors in China and its implications for culturally sensitive nursing care.

**Design:**

A qualitative study employing **thematic analysis** to examine survivors' experiences and coping strategies.

**Methods:**

In‐depth interviews were conducted with Chinese breast cancer survivors to investigate how acts of reciprocity influenced their identity, relationships, and emotional well‐being. Thematic analysis was applied to identify key patterns in their recovery journey.

**Results:**

Repaying kindness enabled survivors to restore disrupted familial roles, uphold cultural values such as **filial piety**, and regain a sense of control post‐diagnosis. This fostered a renewed **sense of purpose**, strengthened **family bonds**, reduced **stigma**, and promoted **psychological resilience**, facilitating identity reconstruction and enhanced social connectedness. These findings highlight the importance **of reciprocity and relational harmony** as transformative coping strategies in cancer survivorship. A culturally informed understanding of recovery suggests that **integrating reciprocal caregiving and family dynamics** into survivorship care can empower patients, enhance resilience, and promote **culturally competent nursing practices** in collectivist societies.

**Patient or Public Contribution:**

Breast cancer survivors contributed to this study by sharing their lived experiences, providing insights into the role **of repaying kindness** in their recovery. Their narratives helped shape the analysis and ensured the findings reflect the realities of coping and resilience in the Chinese cultural context.

## 1 Introduction

Breast cancer is one of the most common malignancies amongst women globally, presenting challenges that extend beyond physical health to include significant emotional and social consequences (Bray et al. 2018; World Health Organization 2021). Recovery involves not only physical healing but also psychological resilience, identity reconstruction, and social reintegration. These dimensions are particularly salient in collectivist societies like China, where family and community are central to individual identity and well‐being (Burgess et al. 2005; Kleinman 1980; Yan et al. 2022).

For Chinese breast cancer survivors, cultural values deeply influence recovery. The diagnosis and treatment often disrupt family roles, body image, and social expectations, leading to psychological distress. Despite these challenges, many survivors exhibit resilience, finding meaning in adversity and engaging in supportive networks to rebuild their lives (Northouse et al. 2002; Zhai et al. 2019; Jin et al. 2021). Family support, shaped by cultural values like filial piety (xiàoshùn), plays a central role in recovery, creating a sense of obligation to repay caregiving efforts and framing recovery as both personal and relational (Cheng et al. 2015; Li and Li 2018). However, much of the existing research focuses on narratives of suffering and obligation, overshadowing active and empowering strategies that survivors use to reclaim agency. Practices like reciprocity and gratitude, integral to Chinese cultural frameworks, are underexplored as mechanisms that foster recovery and psychosocial resilience (Bedford and Yeh 2019; Chen and Li 2012a, 2012b).

In nursing and healthcare, culturally competent care is increasingly recognised as essential for addressing the unique sociocultural needs of patients. Culturally sensitive approaches improve patient outcomes, foster trust, and promote empowerment and resilience (Leininger and McFarland 2006; Andrews and Boyle 2019). One such coping mechanism is **“repaying kindness”** (*bào'ēn*), a culturally embedded practice of expressing gratitude through action—often towards family members, caregivers, or communities. Rooted in Confucian ethics, this practice encompasses both emotional gratitude and moral obligation, shaping survivors' recovery journeys through relational ethics and social reintegration.

This study explores repaying kindness, a practice rooted in Chinese traditional values, emphasising the moral obligation to repay kindness and fostering reciprocity and social harmony (Chen and Li 2012a, 2012b; Bedford and Yeh 2019). This culturally informed framework supports identity reconstruction, emotional resilience, and social reintegration, offering valuable implications for nursing interventions. By incorporating such practices into care, nurses can empower survivors, enhance psychosocial healing, and deliver culturally competent recovery programmes.

## 2 Background

Breast cancer is the most common cancer amongst Chinese women, with over 357,000 new cases and 75,000 deaths reported in 2022 (National Cancer Center 2023). More than 3.75 million women are living with the disease, and although the five‐year survival rate has improved to 82% (Li et al. 2023), rising incidence and survivorship trends reflect an urgent need for culturally tailored post‐treatment care (Zheng et al. 2022). A longitudinal analysis of breast cancer epidemiology in China (Long et al. 2025) underscores this growing burden and calls for deeper attention to survivors' psychosocial and emotional recovery. In China, the recovery experiences of breast cancer survivors are deeply influenced by cultural, familial, and societal factors. While existing research often highlights themes of suffering, fatalism, and filial piety, limited attention has been given to culturally specific and empowering coping mechanisms and culturally sensitive practice. This section examines existing literature to contextualise the recovery experiences of Chinese survivors, emphasising the gaps this study addresses.

### 2.1 Recovery Experiences of Breast Cancer Survivors

Western research highlights more individualised recovery processes, where survivors often rebuild identity through narratives of personal growth and empowerment (Charmaz 1991). However, the recovery of Chinese breast cancer survivors is shaped by a complex interplay of biomedical and sociocultural factors. Studies show that recovery often involves navigating disrupted familial roles, emotional distress, and identity reconstruction (Cheng et al. 2015). Many Chinese women perceive breast cancer as a destabilising event, leading to guilt for being a perceived burden to their families and social withdrawal due to body image concerns, particularly after mastectomies (Zhang, Chen, and Lin 2017; Zhang, Zhang, and Lin 2017).

### 2.2 Cultural Influences on Breast Cancer Recovery

Cultural norms such as filial piety, traditional gender roles, and fatalistic attitudes significantly shape the recovery pathways of Chinese survivors. Filial piety, a cornerstone of Chinese culture, emphasises lifelong obligations to care for parents and elders. Some survivors feel indebted to their parents and express a strong desire to repay this debt, even during their illness (Liu 2010a, 2010b). This dynamic often creates tension between their recovery needs and caregiving responsibilities.

Gender roles further compound these challenges. As primary caregivers in Chinese society, women are often expected to resume caregiving roles soon after treatment, despite ongoing physical or emotional struggles (Cheng et al. 2015). Fatalism also plays a role in shaping recovery. Many Chinese women view cancer as an inevitable fate, fostering resignation and passivity (Zhang, Chen, and Lin 2017; Zhang, Zhang, and Lin 2017; McDonald et al. 2014).

Studies (Chan et al. 2017) indicate that the application of Guolin‐Qigong, a traditional Chinese exercise, can effectively improve the body–mind health of Chinese women with breast cancer. The randomised controlled trial demonstrated significant benefits in physical and psychological well‐being, suggesting that integrating such complementary therapies can enhance holistic recovery approaches.

### 2.3 Culturally Informed Recovery: Moral Obligations and Psychosocial Support

Recent studies underscore the value of culturally informed survivorship care for Chinese breast cancer patients. Li et al. (2022) highlight how traditional gender norms and family expectations shape post‐treatment coping, with moral obligations and relational roles central to emotional adjustment and identity reconstruction. Their findings support this study's emphasis on *repaying kindness* as a culturally meaningful framework for recovery and reintegration.

Similarly, Wang et al. (2025) demonstrate that culturally tailored interventions—such as peer support, family counselling, and psychosocial care—promote resilience by aligning with Confucian values of reciprocity and interdependence. These studies reinforce *repaying kindness* as both a moral logic and a practical basis for designing effective nursing interventions in collectivist settings.

### 2.4 Addressing the Gap: The Role of Repaying Kindness

Existing literature on Chinese breast cancer survivors primarily emphasises themes of suffering and fatalism, with limited attention to empowering coping strategies. The concept of *repaying kindness*—the act of repaying kindness and support—is a culturally embedded mechanism that has been largely overlooked. Rooted in Confucian ethics, *repaying kindness* highlights reciprocity as a moral obligation, providing survivors with a pathway to restore their identity, foster resilience, and rebuild relationships (Bedford and Yeh 2019).

In contrast, Western contexts often frame reciprocity as mutual emotional support or community engagement, lacking the deeply ingrained moral obligations central to Chinese cultural norms (Charmaz 1991; Avis et al. 2005). This cultural distinction underscores the need to explore *repaying kindness* as a uniquely Chinese and empowering coping strategy. While previous studies have acknowledged its role in recovery (Chen and Li 2012a, 2012b), the nuanced dynamics of *repaying kindness*—how it operates and contributes to survivors' psychological and social recovery—remain underexplored. This gap highlights the importance of investigating *repaying kindness* as a culturally grounded framework for understanding breast cancer survivorship in China.

By focusing on repaying kindness, this study shifts the narrative from victimhood to agency, demonstrating how survivors actively engage in reciprocal caregiving and relational harmony as mechanisms for recovery. The findings aim to bridge existing gaps by providing nursing professionals with insights into culturally sensitive care practices, supporting identity reconstruction and holistic recovery for breast cancer survivors in collectivist contexts.

## 3 Methods

This qualitative study aimed to explore the lived experiences. However, the sample—drawn from an urban Beijing‐based organisation and comprising mostly married, employed women with Stage I–III cancer—may limit generalisability. Future studies should include rural, metastatic, or unmarried survivors to reflect more diverse experiences of Chinese women diagnosed with breast cancer, focusing on the sociocultural dimensions of their recovery. Using in‐depth interviews, the study sought to provide a nuanced understanding of how survivors navigate their recovery journeys, with particular attention to the role of *repaying kindness*.

The research team consisted of three members: two female university faculty members with doctoral degrees and one male research assistant with a master's degree. The selection of this topic was informed by the personal experiences of team members who had family members affected by breast cancer, alongside a shared commitment to improving the well‐being of women facing this illness. The study was conducted in collaboration with a breast cancer rehabilitation organisation in Beijing between February 2021 and January 2022.

### 3.1 Sampling

Qualitative research emphasises understanding complex phenomena through detailed exploration rather than statistical generalisation (Creswell and Poth 2018). Studies employing similar methodologies often use sample sizes ranging from 6 to 15 participants for in‐depth interviews (Guest et al. 2006). The chosen sample size of 12 aligns with these established practices and supports the study's objectives, ensuring a diverse representation of demographic and clinical variables such as age, time since diagnosis, marital and fertility status, employment status, and disease stage. This heterogeneity enabled the study to capture a broad range of recovery experiences, enriching the depth and breadth of the findings.

Participants were selected based on the following criteria: (1) voluntary participation, (2) a willingness to share personal experiences, and (3) openness to engaging with researchers. Recruitment was facilitated through the collaborating rehabilitation organisation. Rapport was established through an introductory session where researchers explained the study and engaged in group discussions with potential participants. This approach fostered trust and encouraged candid participation (Table 1).

**TABLE 1:** Personal and medical characteristics of the participants.

Code	Age	Cancer age	Marital status	No. of children	Employment	Tumour stage of diagnosis
Case 1	45	3	Single	0	Employed	II
Case 1	45	3	Single	0	Employed	II
Case 2	41	3	Married	1	Employed	III
Case 3	30	1	Single	0	Quit since cancer	I
Case 4	45	6	Married	2	Employed	IV Secondary recurrence of cancer
Case 5	67	28	Married	1	Volunteer service work	III Secondary recurrence of cancer
Case 6	48	10	Married	1	Employed	III
Case 7	37	2	Married	1	Quit since cancer	III
Case 8	35	4	Single	0	Employed	III
Case 9	39	3	Married	1	Quit since cancer	III
Case 10	40	3	Married	2	Quit since cancer	II
Case 11	69	15	Married	1	Volunteer service work	III Secondary recurrence of cancer
Case 12	45	2	Married	1	Employed	I

### 3.2 Data Collection

Data collection was conducted through semi‐structured interviews guided by an interview protocol. Each interview lasted between 2 and 4 h, with up to three follow‐up interviews for clarification. Interviews were conducted in Mandarin Chinese and focused on: participants' psychological experiences, illness perceptions, the impact of breast cancer on their lives, coping mechanisms, and the role of family and peer support systems.

The interviews were held one‐on‐one at the rehabilitation centre and were audio‐recorded with participants' consent. Detailed field notes were also taken to capture non‐verbal cues and contextual insights. The retrospective nature of the interviews encouraged deep reflection, enabling participants to articulate their recovery journeys comprehensively (Seidman 2013).

### 3.3 Data Analysis

The interview data were transcribed verbatim and subjected to a rigorous, multi‐stage coding process to ensure a reliable and in‐depth interpretation of participants' narratives. The initial phase involved open coding, where the first two authors independently reviewed transcripts to identify key phrases and recurring ideas, generating preliminary codes such as “gratitude towards caregivers” and “desire to repay support.” Collaborative discussions followed to compare and reconcile differences in interpretations, with researchers referring back to the raw data to ensure accuracy. The coding process was structured around thematic analysis (Braun and Clarke 2006), emphasising the identification and interpretation of patterns related to participants' coping strategies and recovery experiences. Through axial coding, related codes were grouped into broader categories, such as combining “volunteering” and “peer mentorship” under the theme “Active Giving Back.” This iterative process also refined the code tree to better capture the relationships and progression between themes, such as the transition from “Gratitude” to “Active Giving Back.”

To ensure reliability, the team engaged in thematic validation discussions and cross‐referenced findings with participant quotes to ground the analysis in the data. Investigator triangulation, involving researchers with diverse academic backgrounds, enriched the interpretation of findings. Reflexive journals documented researchers' assumptions and minimised biases, while member checking allowed participants to validate the themes, ensuring resonance with their lived experiences. This iterative and collaborative approach strengthened the credibility and depth of the analysis, resulting in robust and trustworthy findings.

### 3.4 Rigour

To enhance trustworthiness, the study adhered to the criteria proposed by Lincoln and Guba (1985), ensuring credibility through prolonged engagement with participants and member checking, transferability by providing detailed descriptions of the research context and participant demographics, dependability through an audit trail documenting the research process and analytical decisions, and confirmability by maintaining a reflexive stance and transparency in data interpretation. This rigorous methodology ensured that the findings authentically captured the lived experiences of Chinese breast cancer survivors, offering valuable insights into culturally specific coping mechanisms such as repaying kindness.

### 3.5 Ethics Statement

This study was approved by the ethical committee of REDACTED and followed the Declaration of Helsinki (World Medical Association 2013). Informed consent was obtained, ensuring participants understood the study's purpose, procedures, and risks. Confidentiality and anonymity were safeguarded, and participants could withdraw at any time without consequences.

## 4 Findings

The dynamics of repaying kindness play a crucial role in the social psychological recovery of breast cancer survivors in China. Repaying kindness is not simply a one‐time act of returning a favour but a continuous, evolving process that shapes survivors' recovery through multiple emotional, familial, and cultural dimensions. The dynamics of reciprocity operate across the survivors' emotional responses to cancer, starting with initial fear, social withdrawal, and self‐denial, and evolving into active coping strategies that allow them to reclaim control, reaffirm their identities, and restore family roles. This section elaborates on each theme, highlighting how survivors' initial responses to the diagnosis, such as fear, social withdrawal, and self‐denial, evolve into active coping and identity reconstruction through the process of reciprocity.

### 4.1 Stage 1: Receiving Care

This stage is marked by participants' dependence on family, friends, or peers for emotional, physical, or logistical support during their illness. Participants express feelings of vulnerability, reliance, and gratitude for the care they receive.

#### 4.1.1 Dependency and Vulnerability

In the initial stage following diagnosis, many breast cancer survivors experience profound emotional distress and vulnerability. Survivors often report feeling weak, frightened by coming death, and unable to meet their basic needs.

For me, cancer equals death. I fell apart when that sank in. (Case 11)

I feel like a total burden on my family now, unable to do anything right and just overwhelmed with guilt. (Case 12)

These quotes reflect the dependency survivors feel in the face of a cancer diagnosis, where the care and support they receive become essential for survival. However, this dependency can also bring about a sense of guilt for survivors, who feel they are imposing on their loved ones.

#### 4.1.2 Care and Love From Family Members

During this time, the participants rely heavily on family members, friends, and medical professionals for both physical and emotional support. The vulnerability of this stage is emotionally challenging, as survivors are not only physically weakened but also mentally burdened by the overwhelming fear of death and the uncertainty of treatment.

My husband said life is the most important. With a tumour, cut the breast, the cleaner the better. So I never thought amastectomy was a big problem. I'm not worried about it, either. I believed that I will survive.(Case 5)

During my treatment, I lost my hair. My husband convinced me to shave it off. He then decided to shave his own hair to match mine. We ended up with two baldies at home, and I slowly let go of my hair‐related worries. To live is the goal for me in the coming future. (Case 6)

These statements demonstrate how guilt often accompanies the early stages of illness. Survivors are culturally conditioned to feel indebted to their caregivers, especially in Chinese culture, where the idea of filial piety emphasises the reciprocal obligations between children and parents. As a result, women in this stage not only feel vulnerable but also experience embarrassment or shame for their inability to contribute in return.

#### 4.1.3 Peer Support

Peer support and survivorship programmes create a sense of community and shared experiences, fostering a supportive environment for recovery (Spiegel and Bloom 1989; Stanton et al. 2015). Managing treatment‐related side effects, such as fatigue and pain, is a significant aspect of survivorship, aiding breast cancer survivors in coping (Manne et al. 2005; Ussher et al. 2019).

Social interactions help reduce the stigma associated with cancer (Dujisawa and Hagiwara 2015). By engaging in social interactions, the participants began to regain their belief in life and learned to coexist with cancer. Gradually, their misconceptions about life started to dissipate.

I joined this patient group four months postsurgery. The recovered patients answered our questions, which was crucial. It inspired me to face challenges head‐on and showed me that there's hope beyond the darkness. (Case 4)

I met a woman who has fought for different cancers for many years, I was deeply moved and mobilised by her. She is my role model, I will try to be a person like her. (Case 3)

#### 4.1.4 Indebtedness to Their Family Members

This initial stage of receiving favours is marked by emotional vulnerability, as survivors are deeply aware of their dependency on others. The cultural expectation to eventually repay the care and support they are receiving creates an emotional challenge. Survivors feel an acute sense of indebtedness to their family and caregivers, which makes them emotionally vulnerable. This deep sense of indebtedness is critical to the dynamic of repaying kindness, where the obligation to eventually give back is internalised early on in the recovery journey, setting the foundation for later stages of active coping.

### 4.2 Stage 2: Gratitude and Sense of Obligation

Gratitude is defined as a positive emotional response that arises when individuals recognise and appreciate the kindness, support, or benefits they have received from others (Emmons and McCullough 2003). A sense of obligation refers to a moral or ethical responsibility to repay, reciprocate, or fulfil a duty, often arising from social norms, cultural expectations, or personal values (Bedford and Yeh 2019). In this study, gratitude is understood as the participants' acknowledgment of the support they received from family, friends, and healthcare providers during their illness. Sense of obligation is interpreted as the resulting moral responsibility to repay this support, often manifested in acts of caregiving, community engagement, or familial duties. This transition occurs when participants begin to internalise the support they received and express a desire to reciprocate. Gratitude evolves into a sense of moral responsibility, often influenced by cultural norms like filial piety.

#### 4.2.1 Strong Will to Support Elderly Parents

As the participants received medical treatment, care from their families, and support from peers, their physical conditions improved, prompting them to reflect on their lives through the lens of filial piety—a deeply ingrained cultural value in Chinese society, rooted in Confucian traditions, which emphasises fulfilling one's responsibilities towards parents.

As the eldest in my family, I cannot leave before my mother. Rain or snow, I exercise daily with one belief—to fulfill my duty as the eldest daughter. (Case 5)

Being the only child, my mother expects me to take care of myself, to be by her side always. I cannot let her only child go before her; I must live longer. (Case 7)

#### 4.2.2 Cognition of Family Responsibilities

Also, when they regain energy, they try to pick up their family responsibilities. One participant explained:

I couldn't just sit there and let my family suffer with me. My mother and husband were always so supportive. I promised them that once I was strong enough, I would take care of them the way they cared for me.(Case 12)

It was hard to get up, but I knew I had to get back on my feet. I needed to show my husband and kids that I wasn't going to let cancer defeat me.(Case 9)

This expression of gratitude transforms into an emotional motivation to recover. The obligation to give back becomes a central driver for their psychosocial recovery, fueling their transition from passivity to active coping. Survivors start to engage in behaviours that reaffirm their family roles and restore their identity as caregivers, contributing back to their family's well‐being.

#### 4.2.3 Willingness to Help Others

As part of their recovery journey, many women expressed a strong willingness to help others, driven by their gratitude for the peer support they received. This desire to give back often serves as a way to pay forward the kindness and assistance they experienced during their most challenging times.

Several participants articulated their intention to provide help to others in the future, emphasising the significance of peer support in fostering hope and resilience. For example, one participant shared:

If I can be cured, I will do volunteer work, just like my peer patients did for me. They were so kind and helpful to me when I was at my lowest point. (Case 6)

This sentiment reflects how the experience of receiving care motivates survivors to contribute to their community, transforming their gratitude into meaningful action and reinforcing the cycle of support within their social networks.

The evolving sense of gratitude and moral obligation is closely tied to the dynamic of repaying kindness. Gratitude moves beyond emotional relief to a genuine desire to reciprocate the support. The motivation to recover is no longer just for survival but to fulfil familial responsibilities. This transformation, driven by the desire to give back, becomes a core emotional motivator in their recovery process, moving the survivors into a phase of active coping and identity reconstruction.

### 4.3 Stage 3: Active Giving Back

This stage is defined by concrete actions where participants actively engage in caregiving or volunteer work. They shift from being passive recipients to active contributors in familial or social contexts. Participants describe specific acts of giving back, such as providing emotional support to peers, volunteering in cancer support groups, or resuming caregiving roles within their families. Many women report that the act of giving back—whether through taking care of children, providing emotional support to spouses, or helping ageing parents—is integral to their recovery and identity restoration.

#### 4.3.1 Regaining a New Identity

##### 4.3.1.1 Positive Identity Reconstruction Through Role Models

Peer group involvement and interactions with inspiring figures—such as fellow survivors, healthcare providers, and community leaders—play a crucial role in the positive reconstruction of identities for women recovering from breast cancer. These interactions help women shift their self‐narratives from a focus on survival to living purposefully with hope for the future.

For example, one participant reflected:

I met a fellow survivor who had both breast and colon cancer but didn't let her diet be restricted. She has been cancer‐free for years. It made me rethink my worries and start socialising again. (Case 8)

Others expressed how witnessing the resilience of peers inspired their own recovery journeys:

Seeing someone like me who went through this and came out stronger helped me believe I could do the same. (Case 12)

Drawing inspiration from role models allows women to reshape their self‐perceptions, moving away from fear and helplessness towards empowerment and a renewed sense of agency. These experiences motivate them to reclaim control over their lives and actively participate in their recovery.

##### 4.3.1.2 Living Harmoniously With Cancer

Support from peers and groups also facilitates the development of coping strategies that help women coexist peacefully with their cancer. By engaging with others who share similar experiences, they gradually downplay the severity of their condition, find psychological balance, and assign new meaning to their illness. Participants described this transformative process:

I have started accepting my disease. The prognosis is good, and with active treatment, I believe I will overcome it. (Case 2)

I see this as a chronic condition. Once I adapt, anxiety lessens. I'm learning to live normally. (Case 4)

Through these experiences, women learn to redefine their illness as part of their life journey rather than an impending threat. This redefinition empowers them to embrace cancer as a manageable condition, reducing anxiety and enabling them to focus on living a fulfilling life.

Ultimately, the support of peers and role models catalyses a profound transformation in how women perceive themselves and their illness. They replace fear with resilience, redefine their identities, and find a sense of purpose that drives their recovery journey.

#### 4.3.2 Reclaiming Family Roles Through Giving Back

As survivors progress in their recovery, they begin to reaffirm their roles within the family, shifting from a position of vulnerability to one of reciprocity. The moral obligation to repay kindness (especially for the care and support they received from family members) becomes a key motivator in their recovery journey. Many survivors feel empowered by the desire to restore family roles—particularly as mothers, wives, and daughters.

As Foster, Breckons, and Cotterell (2012) and Foster, McDonald, and Newell (2012) suggest, giving back not only serves as a mechanism for emotional healing but also helps survivors reaffirm their family responsibilities. The act of reciprocity (repaying kindness) enables them to move from helplessness to empowerment as they reconnect with their family roles.

Now that I am better, I cook for my children again. It feels so good to be able to do the little things for them, to show them that I'm still here, still strong. (Case 4)

It felt amazing when I was able to help my mother after everything she did for me. It was like I was finally able to pay her back. (Case 6)

This shift marks the dynamic evolution of repaying kindness from a passive receipt of care to active caregiving. Survivors' willingness to repay is driven by an internalised obligation to restore family harmony and fulfil cultural expectations. This not only strengthens family ties but also enhances survivors' self‐worth and social integration. Giving back allows survivors to reconstruct their identity as resilient, capable family members.

#### 4.3.3 Fulfilling Filial Duties Through Reciprocity

As survivors begin to recover, repaying kindness becomes an important strategy to reaffirm filial obligations and restore emotional balance. By giving back—whether through caring for ageing parents, supporting spouses, or fulfilling family responsibilities—survivors feel they are rebalancing the family dynamic. This active engagement in reciprocal caregiving helps alleviate guilt and allows survivors to restore their familial roles.

As Liu (2010a, 2010b) explained, the act of giving back within the family not only restores moral order but also helps survivors regain their social legitimacy as daughters, mothers, or wives.

Now that I am better, I help my parents with their daily activities. It feels right to do this. It helps me feel like I am fulfilling my duty as a daughter. (Case 4)

I made sure my husband didn't feel like he was taking care of everything alone. I wanted to give back to him for all the support he had given me. (Case 7)

This shift marks the dynamic transition from passive dependence to active caregiving, where the dynamic of repaying kindness truly unfolds. Survivors begin to repay the care and support they received, fulfilling cultural expectations that reaffirm their role within the family and alleviate feelings of guilt. Through this process of giving back, they reclaim their position as caregivers, contributing to their psychosocial recovery. Active participation in family life provides psychological empowerment and restores self‐worth, reflecting the cultural imperative to repay care. This central aspect of their identity reconstruction fosters a sense of empowerment and resilience during the recovery journey.

### 4.4 Stage 4: Social Engagement

This stage marks a significant expansion of reciprocity, as survivors extend their gratitude beyond immediate family to broader societal contributions. By redefining their roles within their communities, they foster a sense of belonging and social purpose. For many, returning to normal life involves taking on roles that enable them to give back, not only to their families but also to society. This process reflects their appreciation for the support received, facilitates psychosocial recovery, and promotes personal growth, fulfilling the moral obligation of requiting favours.

#### 4.4.1 Reintegrating by Going Back to Work

The workplace emerges as a critical space for survivors to redefine their identities and expand their circle of reciprocity. Support from employers, supervisors, and colleagues provides an opportunity for survivors to transition from feelings of inadequacy to rediscovering their strengths.

Upon returning to work, my colleagues interacted with me naturally, avoiding mentioning my illness and treating me like any other person. This made me feel comfortable and accepted. (Case 2)

Despite knowing about my breast cancer, my boss sought my input and assigned me a team to lead, treating me as a normal person. Feeling trusted, I became more motivated at work, and my overall mood improved. (Case 8)

By reintegrating into the workforce, survivors feel valued and empowered. This positive reinforcement not only strengthens their professional identity but also provides a platform to contribute meaningfully to society, fulfilling their sense of reciprocity.

#### 4.4.2 Reconnecting to Public Life

Requiring favours also extends to public interactions, where women re‐enter social spaces with a renewed sense of belonging. As they experience acceptance and care from the public, their previous fears of stigma and rejection are replaced with a deeper understanding of societal goodwill.

I realised that when I explained to the salesperson about my condition, they were not judgmental but rather very helpful. Now I confidently inform them about my breast cancer and try out underwear in the fitting rooms. (Case 4)

Once my friends learned about my diagnosis, they were supportive, suggesting traditional Chinese medicine and sharing dietary therapy tips with me. (Case 10)

Positive public interactions challenge survivors' preconceived notions of judgement and gossip, helping to dismantle internalised stigma. These moments of reciprocity, where the public provides care and understanding, empower survivors to embrace social life with confidence, allowing them to feel connected and supported.

#### 4.4.3 Bringing Meanings to Life by Engaging in Volunteer Work

Engaging in volunteer work is a meaningful way for these women to repay kindness they received from society, particularly the support and encouragement from peer volunteers. They basically participated in the volunteer work after their diagnosis. Grateful for the assistance they experienced during their own recovery, they actively participate in support groups and volunteer to help new patients, sharing their experiences and offering guidance to others who understand their journey. These acts of giving back not only foster connections with individuals who have undergone similar challenges but also play a vital role in their psychosocial recovery. By reducing feelings of isolation and providing emotional validation, these women find a renewed sense of purpose and recognise their value in helping others. Through these experiences, they imbue their lives with new meaning, transforming their journey into a source of strength and solidarity.

Now, I take volunteering as my job for the rest of my life. I think helping others make me feel more valuable, and happy. I go to the self‐help groups, meeting different sisters, talking to them, and doing exercises and singing, dancing with them. (Case 5)

As a volunteer in the organisation, I feel so proud, that I survived, and I can talk to different people there, and share and learn from them. I guess other volunteers will have similar feeling too. (Case 11).

Through reintegration into work and public life, survivors actively transform gratitude into tangible actions, contributing to society through professional roles or social engagement. These acts foster psychosocial recovery, instil a renewed sense of purpose, and help survivors reclaim their place in society. By giving back, they find new meaning in life, reflecting the cultural significance of repaying kindness in their recovery journey.

The social engagement phase extends this dynamic beyond the family to the broader community. Survivors often feel a moral responsibility to support others, particularly fellow cancer patients, through advocacy, volunteerism, or sharing their stories. These efforts reinforce their sense of agency, social empowerment, and the reciprocal cycle of care, further enriching their recovery process.

### 4.5 Stage 5: Emotional and Psychological Benefits of Repaying Kindness

The final stage is characterised by participants reporting feelings of empowerment, fulfilment, and emotional resilience. They demonstrate a reconstructed sense of identity and acceptance of their illness as part of their life journey. Participants describe improved mental well‐being, a stronger sense of self, and a shift from viewing cancer as a burden to seeing it as a source of personal growth.

The act of requiting favours through caregiving, volunteering, and social engagement plays a transformative role in breast cancer survivors' recovery. By giving back, survivors experience profound emotional and psychological benefits, which consolidate their healing process and empower them to redefine their lives.

#### 4.5.1 Empowerment

By transitioning from recipients of care to contributors, survivors reclaim control over their lives and rediscover their sense of agency as well as usefulness. This empowerment allows them to overcome feelings of helplessness and recognise their capacity to make a meaningful impact.

When I started volunteering, I felt like I wasn't just a cancer survivor but someone who could still contribute meaningfully. (Case 11)

“Through giving back, I felt like I became whole and independent again, not just someone battling cancer.” (Case 9)

Every time I saw a smile on another patient's face because of my help, I felt fulfilled and proud. It was as if my life had meaning again. (Case 8)

Acts of reciprocity, such as volunteering or supporting family members, provide survivors with a sense of accomplishment and renewed purpose. These experiences allow them to see their value and contributions, bringing joy and satisfaction to their lives.

#### 4.5.2 Identity Reconstruction

Survivors redefine their self‐perception, moving beyond the identity of a patient to become active contributors in their families and communities. This process helps them regain their sense of self‐worth and reconstruct their identities as resilient and capable individuals.

Helping my husband with daily chores gave me a sense of control over my life again. It made me realise I was no longer dependent but capable of helping others. (Case 7)

I used to see myself as weak and dependent, but now I see myself as someone strong who can support others. (Case 12)

#### 4.5.3 Resilience

Positive interactions and acts of reciprocity strengthen survivors' psychological resilience, equipping them to face future challenges with optimism and determination. These experiences help survivors develop coping mechanisms and view their journey as a source of strength.

I learned to handle setbacks with a positive attitude because I know I am capable of overcoming them. Helping others reminded me of my own strength. (Case 2)

I went through my brush with the death, and I survived, from now on, I will never fear of any difficulties in life. (Case 9)

The emotional and psychological benefits of repaying kindess—empowerment, fulfilment, identity reconstruction, and resilience—are key milestones in breast cancer survivors' recovery journey. Through giving back, survivors transform their sense of gratitude into meaningful actions, enabling them to reclaim their agency, rebuild their lives, and emerge stronger than before. These experiences not only help survivors heal but also inspire them to contribute to the well‐being of others, creating a powerful cycle of support and resilience.

Table 2 summarises the five key stages of recovery, each with related subthemes. The “Ranking” column indicates how many of the 12 participants mentioned each subtheme, reflecting its prevalence across cases.

**TABLE 2:** Themes and subthemes of the findings.

Stages/Themes	Subthemes	Ranking
1. Receiving care	Dependency and vulnerability	8/12
	Care and love from family members	11/12
	Peer suppor	9/12
	Indebtedness to family members	11/12
2. Gratitude and Obligation	Strong will to support elderly parents	6/12
	Cognition of family responsibilities	9/12
	Willingness to help others	11/12
3. Active giving back	Positive identity reconstruction	9/12
	Living harmoniously with cancer	10/12
	Reclaiming family roles	9/12
4.Social engagement	Reconnecting to public life	8/12
	Volunteer work and meaning making	11/12
5.Psychological benefits	Empowerment	7/12
	Identity reconstruction	9/12
	Resilience	7/12

## 5 Discussion

### 5.1 Pathways of Repaying Kindness

**FIGURE 1:**
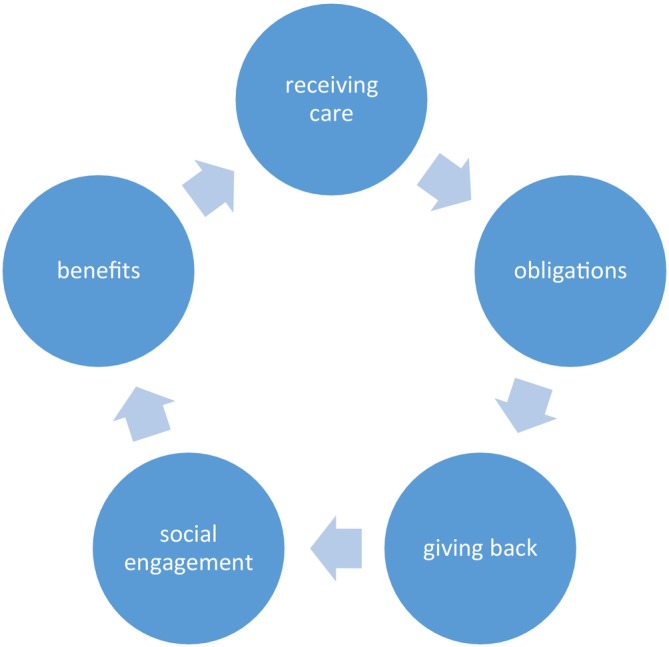
Pathways of repaying kindness.

This study delineates five interconnected stages of *repaying kindness*, emphasising their cultural significance in the recovery journeys of Chinese breast cancer survivors as shown in Figure 1:

- *Phrase 1: Receiving care as the foundation*: The initial reliance on family and peers establishes a moral obligation to repay, forming the basis of reciprocal recovery.
- *Phrase 2: Gratitude and obligation as motivators*: Gratitude transforms into a culturally informed pathway for recovery, grounded in Confucian values such as filial piety.
- *Phrase 3: Active giving back*: Survivors transition to active caregiving, restoring familial balance and reinforcing moral and social order.
- *Phrase 4: Social engagement*: Survivors expand their reciprocity beyond familial boundaries to societal contributions, fostering a sense of belonging and purpose.
- *Phrase 5: Emotional and psychological benefits*: Empowerment, fulfilment, and resilience emerge as survivors complete the recovery cycle, highlighting the cultural framework of reciprocity as a source of healing.

These pathways present a culturally specific paths to recovery, contrasting with individualistic models frequently discussed in Western literature. For instance, Charmaz (1991) highlights self‐reliance and personal growth as central to recovery in Western contexts. In contrast, this study underscores the interconnectedness and reciprocal obligations that drive recovery in a collectivist culture, where interdependence and mutual support are integral.

### 5.2 Contributions to Existing Literature

This study makes significant contributions to the understanding of breast cancer recovery by introducing *repaying kindness* as a dynamic, culturally embedded coping mechanism. It highlights how the act of repaying kindness transforms survivors' recovery journeys into active processes of reciprocity, fostering psychological resilience, identity reconstruction, and social reintegration.

#### 5.2.1 Repaying Kindness as an Active Coping Mechanism

Unlike prior research that focuses on the psychosocial challenges of breast cancer recovery (Foster, Breckons, and Cotterell 2012; Foster, McDonald, and Newell 2012; Liu 2010a, 2010b), this study demonstrates how *repaying kindness* motivates survivors to transition from passive recipients of care to active contributors within their families and communities. Survivors engage in caregiving, volunteering, or mentoring, which enhances their self‐worth, reduces feelings of isolation, and shifts their focus from illness to the positive impact of their actions. This approach contrasts with narratives emphasising fatalism or stigma in earlier studies (Zhang, Chen, and Lin 2017; Zhang, Zhang, and Lin 2017). This builds on Li et al.'s (2022) findings that Chinese women often seek emotional healing by fulfilling moral roles and restoring family responsibilities, positioning relational duty as a pathway to recovery.

#### 5.2.2 Filial Piety as a Pathway to Recovery

The study reinterprets filial piety, a core Confucian value, as a positive force in recovery. Survivors often take on caregiving roles for ageing parents or family members, drawing strength and purpose from these culturally meaningful duties. While prior research has emphasised filial piety's restrictive aspects (Cheng et al. 2015; Liu 2010a, 2010b), our findings suggest it can foster resilience when voluntarily embraced. Rather than a burden, fulfilling filial roles becomes a source of agency and psychosocial healing, consistent with Confucian ethics, which view moral identity as grounded in relational responsibility.

#### 5.2.3 Reciprocity and Social Support

This study builds on existing research on peer and family support in cancer recovery (Manne et al. 2005; Stanton et al. 2015) by showing how *repaying kindness* fosters a culturally grounded cycle of reciprocity. Survivors not only receive care but actively give back, which enhances resilience and restores social roles. Wang et al. (2025) highlight that culturally adapted peer support promotes emotional strength through mutual aid and moral exchange—an insight our study deepens through Confucian ethics, where reciprocity is a moral expectation. These findings also extend Liu's (2010a, 2010b), Foster, Breckons, and Cotterell (2012), and Foster, McDonald, and Newell (2012) work by showing how identity is rebuilt through giving, not just receiving.

#### 5.2.4 A Framework for Psychological Empowerment

This study provides a culturally grounded framework for psychological empowerment, emphasising active coping strategies. Through *repaying kindness*, survivors redefine themselves as resilient, empowered individuals. Their contributions to others enable them to transcend the stigma or helplessness often associated with cancer recovery (Foster, Breckons, and Cotterell 2012; Foster, McDonald, and Newell 2012). Acts of reciprocity support identity reconstruction, helping survivors reclaim a sense of purpose and agency disrupted by illness.

These findings not only enrich the existing literature on breast cancer survivorship but also provide actionable insights for culturally sensitive care practices, emphasising the importance of reciprocity, family dynamics, and empowerment in holistic recovery.

#### 5.2.5 Repaying Kindness as Moral Reciprocity: Theoretical and Cross‐Cultural Insights

The concept of *repaying kindness* in this study reflects a culturally embedded form of reciprocity, not merely as behavioural exchange but as a moral and relational imperative. While social exchange theory (Blau 1964) emphasises reciprocity and negotiated trust, our findings show that survivors give back not to balance accounts but to meet ethical obligations rooted in Confucian values of interdependence, role‐based duty, and relational harmony.

This moral logic is evident in survivors' caregiving, peer mentorship, and volunteerism—actions viewed not as burdens but as essential to self‐worth and recovery. Li et al. (2022) noted that Chinese survivors find healing through restoring familial roles, while Wang et al. (2025) showed that peer support grounded in mutual aid enhances psychological resilience. These findings suggest that giving back is central to recovery in Chinese contexts.

In contrast to Western models such as Charmaz's (1991) identity reconstruction—where recovery centres on self‐narration and autonomy—Chinese survivors rebuilt identity through fulfilling expected roles, reaffirming their place in a moral community. This cultural divergence highlights how Chinese models embed resilience in relational continuity and ethical obligation.

Thus, recovery in collectivist settings involves reestablishing moral order and social connection. By integrating social exchange theory with Confucian ethics, this study offers a culturally specific lens on empowerment – grounded in reciprocity, interdependence, and meaningful contribution – offering valuable guidance for relationally attuned nursing care.

### 5.3 Implications for Future Nursing Practice

The findings of this study provide practical insights for nursing professionals working with breast cancer survivors, particularly in collectivist cultures like China. Understanding the role of repaying kindness in recovery allows nurses to better address patients' emotional and psychological needs. Nurses can recognise and support survivors' motivations to engage in reciprocal caregiving, helping them restore familial roles and regain a sense of purpose.

By fostering family‐centerd care, nurses can create supportive environments where survivors and their families collaborate to enhance recovery. Additionally, integrating culturally sensitive practices into care plans, such as encouraging peer mentorship and community engagement, can empower survivors to transition from passive recipients of care to active contributors.

Training programmes for nurses should emphasise the importance of cultural dynamics in shaping recovery experiences, equipping them to deliver holistic, patient‐centred care that aligns with the values and coping mechanisms of diverse populations. While *repaying kindness* is often a source of emotional resilience and moral fulfilment, it can also generate psychological stress when survivors feel compelled to give back beyond their emotional or physical capacity. For instance, Case 12 expressed guilt and self‐blame for not being able to reciprocate her husband's support due to fatigue and ongoing treatment side effects. This underscores the tension between empowerment and obligation inherent in culturally embedded practices like *bào'ēn*.

It is crucial for nurses and healthcare professionals to assess whether patients' expressions of reciprocity are voluntary and restorative, or whether they stem from societal expectations that may intensify distress. Culturally competent care should recognise *repaying kindness* not only as a strength but also as a potential burden, and help survivors find balance between giving back and protecting their own well‐being. Interventions should be sensitive to survivors' personal capacities and offer alternative ways to express gratitude that do not compromise their recovery. These approaches not only enhance emotional resilience and psychosocial healing but also promote a more inclusive and culturally competent healthcare system.

Future research should examine the application of repaying kindness across diverse cultural contexts to identify universal and culture‐specific recovery pathways. Longitudinal studies can assess the long‐term impact of these practices on survivors' mental health and community reintegration. Expanding this framework to other chronic illnesses could further its relevance to nursing care.

By adopting culturally competent approaches, nursing professionals can empower survivors, foster resilience, and deliver holistic, patient‐centred care tailored to diverse sociocultural needs.

## 6 Conclusion

This study underscores the significance of the framework of repaying kindness in understanding the recovery experiences of Chinese women with breast cancer. Rooted in cultural values like filial piety and reciprocity, repaying kindness shifts the narrative from passive coping to active engagement in identity reconstruction, emotional resilience, and social reintegration, offering a culturally informed perspective on recovery in collectivist contexts.

Repaying kindness plays a vital role in restoring survivors' identities by enabling them to reaffirm familial and social roles through reciprocal acts such as caregiving. This fosters a renewed sense of purpose and agency. Additionally, by focusing on their ability to contribute to others, survivors build emotional resilience, reducing feelings of helplessness and strengthening psychological well‐being. The framework also enhances family bonds, as survivors actively reciprocate care, deepening emotional connections and fostering a supportive recovery environment. Furthermore, it facilitates social reintegration by encouraging survivors to participate in community activities, rebuild confidence, and alleviate feelings of isolation.

This culturally grounded framework provides valuable insights for nursing professionals, highlighting the need for interventions that empower survivors to restore familial roles, foster resilience, and reintegrate into their communities. Future research should explore the broader applicability of repaying kindness to develop holistic, culturally sensitive care models for cancer survivors.

## Author Contributions

Conceptualisation and methodology by M.L. and W.L. Data collection by W.L. and M.L. Data transcription by W.L. and X.C. Coding and concept development by W.L., M.L. and X.C. First draft by M.L., W.L. and X.C. Finalised by M.L. and W.L. All authors have made contributions to the research and manuscript.

## Conflicts of Interest

The authors declare no conflicts of interest.

## Data Availability

The data that support the findings of this study are available on request from the corresponding author. The data are not publicly available due to privacy or ethical restrictions.
